# Characterization of IgE cross-reactivity and allergenicity of peanut allergens

**DOI:** 10.1093/immhor/vlaf018

**Published:** 2025-05-30

**Authors:** Christian Lapitan, William R Zhang, Beichu Guo, Tracy R Daniels-Wells, Manuel L Penichet, Ke Zhang

**Affiliations:** Allerdia Inc., Torrance, CA, United States; Division of Dermatology, Department of Medicine, David Geffen School of Medicine at UCLA, University of California, Los Angeles, Los Angeles, CA, United States; Allerdia Inc., Torrance, CA, United States; Division of Surgical Oncology, Department of Surgery, David Geffen School of Medicine at UCLA, University of California, Los Angeles, Los Angeles, CA, United States; Division of Surgical Oncology, Department of Surgery, David Geffen School of Medicine at UCLA, University of California, Los Angeles, Los Angeles, CA, United States; Department of Microbiology, Immunology, and Molecular Genetics, David Geffen School of Medicine at UCLA, University of California, Los Angeles, Los Angeles, CA, United States; Jonsson Comprehensive Cancer Center, UCLA Health, Los Angeles, CA, United States; Molecular Biology Institute, University of California, Los Angeles, Los Angeles, CA, United States; Allerdia Inc., Torrance, CA, United States

**Keywords:** Ara h 2 IgE, IgE cross-reactivity, peanut allergens, peanut allergenicity, peanut anaphylaxis

## Abstract

IgE cross-reactivity among peanut allergens is controversial, and allergenicity of peanut allergens other than *Arachis hypogaea* 2 [Ara h 2] remains to be elucidated. We investigated the origins of peanut IgE cross-reactivity using Western blotting, and allergenicity of peanut allergens employing a passive cutaneous anaphylaxis model. Peanut allergic IgE bound to a large swath of peanut proteins including Ara h 2, Ara h 1, Ara h 3, and Ara h 6. IgE cross-reactivity among peanut allergens could be inhibited by recombinant Ara h 2. Affinity-purified Ara h 2 IgE reconstituted broad IgE binding patterns to Ara h 1, Ara h 3, and Ara h 6 in addition to Ara h 2. Monoclonal human IgE and mouse IgG against peanut allergen component variably bound to other peanut allergen components. Ara h 2 and Ara h 6 could trigger Ara h 2 IgE-mediated peanut allergic reactivity, whereas Ara h 1 and Ara h 3 failed to do so. Ara h 1 IgE was incapable of mediating Ara h 1–triggered allergic reaction. These results revealed that Ara h 2 IgE was the origin of IgE cross-reactivity, and Ara h 2 IgE-mediated peanut allergic reactivity triggered by Ara h 2 and Ara h 6. Ara h 1 and Ara h 3 did not display detectable allergenicity. These results indicated that Ara h 2 IgE appeared to be the “master” responsible for IgE cross-reactivity among peanut allergens and might be the only IgE responsible for allergic reactivity in peanut allergy.

## Introduction

Peanut allergy (PNA) is the leading cause of severe food allergies, affecting 1.5% to 2% of the U.S. population, and can frequently result in life-threatening anaphylaxis.^1–4^ Peanuts (*Arachis hypogaea* [Ara h]) contain at least 18 components capable of binding IgE of patients with PNA^5^; therefore, these IgE-binding components potentially are peanut allergens. Up to date, only a set of components, including Ara h 1, 2, 3, 6, 8, and 9, are thought relevant peanut allergens that are included in the PNA diagnostic panel, allergenicity of other IgE binding peanut proteins remain unclear.

Ara h 2 (a 2S albumin), composed of approximate 10% of total peanut protein contents, have been firmly defined as the most potent peanut allergen component triggering anaphylactic PNA,^6–8^ whereas Ara h 8 (a Bet v 1-like protein) and Ara h 9 (a nonspecific lipid transfer protein) are peanut allergens primarily associated with mild allergic reactions and oral allergy syndrome, rather than with severe anaphylaxis; therefore, Ara h 8 and Ara h 9 are generally considered less significant than Ara h 2 and Ara h 6 in PNA pathogenesis.^5^ Allergenicity of Ara h 1 (a vicilin protein, composed of ∼20% of total peanut protein content), Ara h 3 (a legumin protein), and Ara h 6 (also a 2S albumin), however, are not well defined, and the role of these allergens in PNA pathogenesis remains unclear as monosensitization of Ara h 1–, 3–, and 6–specific IgE have rarely been found and cosensitization of Ara h 2 IgE with IgE to these allergens confounds the relative contributions of Ara h 1–, 3–, and 6–specific IgE in PNA pathogenesis. Due to their heat stability, digestion resistance, and IgE binding capacity,^5^^,^^9^ it is generally believed that Ara h 1, Ara h 3, and Ara h 6 are also the major peanut allergens capable of triggering peanut allergic reactions contributing to anaphylactic PNA, although they are generally considered less potent than Ara h 2.^5^

IgE cross-reactivity among the major peanut allergen components has been previously observed.^10–14^ IgE cross-binding between Ara h 2 and Ara h 6 was thought due to their sequence homologies/structural similarities.^12–14^ However, the basis for IgE cross-reactivity among Ara h 1, Ara h 2, and Ara h 3^9–11^ is not clear, as these peanut allergens share only a low-level sequence homology. A recent study attributed IgE cross-reactivity between Ara h 1 and Ara h 2 to cross-contamination of Ara h 1 preparation with trace amounts of Ara h 2 and/or Ara h 6, arguing against the existence of true IgE cross-reactivity between Ara h 2 and Ara h 1.^15^

Peanut allergen allergenicity and peanut IgE cross-reactivity issues remain puzzled after decades of efforts trying to understand the basis and mechanisms involved. For example, it is not clear why multiple types of peanut IgE always coexist in peanut allergic subjects, whereas monosensitization of any Ara h-specific IgE has been rarely found in PNA patients even though Ara h 1, Ara h 3, and Ara h 6 are thought to be the major peanut allergens. To gain better understanding of these questions, we characterized the nature of IgE cross-reactivity using a modified Western blotting (WB) approach for direct visualization of IgE cross-reactivity and defined the allergenicity of peanut allergen components employing an animal challenge model to examine allergic triggering potency of several major peanut allergen components.

## Materials and methods

### Peanut allergic serological samples

PNA and non-PNA serology (serum or plasma) samples (Table 1) were obtained from Plasmalab International (www.plasmalab.com) with local institutional review board approval. The criteria for PNA samples used in this study included: PNA history ≥2 yr; and PNA diagnosed by physician with peanut IgE (F13 IgE) ≥50 kU_A_/L or Ara h 2 IgE >10 KU_A_/L. The criteria for non-PNA used as control samples are no PNA history with F13 IgE ≥5 kU_A_/L.

**Table 1.** Demographic characteristics of PNA and non-PNA serological samples used in the study.

Sample ID	Sex	Age (yr)	Clinical diagnosis	IgE (KU_A_/L)					
				Total	F13 (peanut)	F422 (rAra h 1)	F423 (rAra h 2)	F424 (rAra h 3)	F442 (rAra h 6)
**PL14231**	**M**	**36**	**PNA**	**ND**	**>100**	**57.42**	**40.63**	**18.2**	**ND**
**PL23064**	**M**	**31**	**PNA**	**538.094**	**92.54**	**28.46**	**51.82**	**4.56**	**7.62**
**PL20264**	**M**	**29**	**Allergic rhinitis, PNA**	**116.435**	**46.88**	**28.31**	**34.66**	**ND**	**8.34**
**PL27861**	**F**	**23**	**Allergic rhinitis, PNA**	**264.742**	**50.46**	**40.12**	**19.42**	**7.61**	**5.27**
**PL24252**	**M**	**52**	**Asthma, eczema, PNA**	**1342.7**	**>100**	**ND**	**>10**	**ND**	**ND**
**PL22744**	**F**	**30**	**Allergic rhinitis, PNA**	**518.645**	**140.6**	**ND**	**>10**	**ND**	**ND**
**PL23861**	**M**	**28**	**Allergic rhinitis, PNA**	**17216.5**	**69.89**	**ND**	**17.37**	**ND**	**ND**
**PL22194**	**F**	**45**	**PNA**	**1392.212**	**342.67**	**72.86**	**161.08**	**ND**	**ND**
**BD2475**	**M**	**59**	**Allergic rhinitis, non-PNA**	**ND**	**15.41**	**ND**	**ND**	**ND**	**ND**

**F, female; M, male;** ND, not determined.

### Purified natural and recombinant peanut allergens and anti-peanut allergen component monoclonal antibodies

The purified natural peanut allergen Ara h 1 (nAra h 1), purified natural Ara h 3 (nAra h 3), *Pichia pastoris***–**derived recombinant Ara h 8 (rAra h 8), rAra h 9, human Ara h 1 IgE E-3B10, Ara h 2 IgE E-11F10, Ara h 3 IgE E-3C3, Ara h 6 IgE E-7B6,^16^ mouse monoclonal antibody (mAb) anti-Ara h 1 2F7, anti-Ara h 2 1C4 and 2B6, anti-Ara h 6 3B8, and anti-Ara h 3 1E8 were purchased from InBio. Mouse mAb anti-Ara h 2 15H4 and CD2 were produced by GeneScript through a service contract using purified nAra h 2 as an immunogen. The chimeric mouse-human Ara h 1 IgE AH1-16D11 and Ara h 2 IgE AH2-2G2 was purchased from AllerMabs. *Escherichia coli*–derived rAra h 1.0101 was purchased from antibodies-online.com.

### Crude peanut extract preparation

Peanut powder (2.3 g, PB&Me; X0012S5CZD) was extracted with 45mL of 0.1 M Tris-HCl buffer pH 7.9 containing NaN_3_ at 0.05% overnight at room temperature with rocking at speed of 20/min. The extracted solution was centrifuged at 4,337 *g* for 60 min at 4 °C to remove insoluble debris and oil layer, the resulted solution was used as crude peanut extract (CPE). Protein concentration of CPE was measured using NanoDrop One (Thermo Fisher Scientific).

### WB analysis of peanut protein–reactive IgE

CPEs (10 µg/lane), under both nonreducing (NR) and reducing (R) conditions (20 mM dithiothreitol, 4% sodium dodecyl sulfate in loading buffer and boiled at 100 °C for 10 min), were fractionated in 10% to 20% gradient Tris-glycine gel (Novex, XP10201BOX; Thermo Fisher Scientific) with standard sodium dodecyl sulfate–polyacrylamide gel electrophoresis conditions (0.1% sodium dodecyl sulfate, 3.03 g/L Tris, 14.44 g/L glycine). The gels were electrophoresed at a constant voltage of 150 V for 80 min (Power Ease 300 W, mini Gel Tank A25977; Invitrogen). Proteins were transferred onto a polyvinylidene difluoride membrane (Immobilon-P; IPVH00010) using Bio-Rad’s Trans-Blot SD Semi-Dry Transfer Cell (221BR) at a constant voltage of 15 V for 20 min. Before blocking, the membrane was washed in phosphate-buffered saline (PBS) for 10 s and allowed to dry for 30 min. The membrane was then rehydrated in PBS-Tween 20 (PBST, Sigma-Aldrich; P3563) for 10 s and the remaining free binding sites were blocked with PBST containing 10 mg/mL soy protein solution. The stock soy protein solutions were prepared by adding 50 g of soy protein powder (NOW Sports soy protein isolate) to 500 mL of PBST stirring with a magnet bar for >2 h, centrifugating for 20 min at 4,337 *g* to remove the insoluble debris, and measuring the protein concentration with NanoDrop One. After ≥1 h of blocking at room temperature (RT), the membrane was cut into strips (each under both NR and R conditions). Each strip was incubated overnight at 4 °C with 1:10 diluted PNA (or up to 1:20 diluted for some samples) and non-PNA samples in soy protein solution, followed by washing with PBST 3 times for 5 min each. The washed blots were incubated with a goat-anti-human IgE conjugated to horseradish peroxidase (HRP) (1:1,000 [vol/vol]; Life Technologies; A18793) in soy protein solution for 1 h at RT, followed by washing with PBST 6 times for 5 min each. WB signal was detected with Bio-Rad’s Clarity Western ECL Substrate (1705061) using the Invitrogen iBright FL 1000 imaging system.

For WB comparing IgE signal patterns from the pre– and post–Ara h 2 affinity chromatography absorption, the total protein concentration of each diluted serum of the pre- and postaffinity absorption were measured and adjusted to equal protein contents for each pair of samples before incubation with the blocked blots.

For WB analysis of human IgE mAbs binding to the recombinant or purified natural peanut allergen components, 0.1 to 1 µg/lane of protein was used under NR conditions, followed by the WB protocol described previously probing with the anti-Ara h 2 human IgE mAb (100 IU/mL), and detecting with anti-human IgE-HRP.

For WB analysis of mouse mAb anti-Ara h 1, anti-Ara h 2, anti-Ara h 3, and anti-Ara h 6 binding to the recombinant or purified natural peanut allergen components, the diluted mAb (1 µg/mL) was used for probing, followed by detecting with anti-mouse IgG-HRP.

### Recombinant peanut allergen construction and expression

rAra h 2 and rAra h 6 were expressed and produced in-house. The synthesized DNA sequences coding for mature Ara h 2.0201 isoform (453 bp DNA fragment coding for 151-amino-acid protein with molecular weight (MW) 19 kDa, GenBank accession number AAN77576) and Ara h 2.0101 isoforms (417 bp DNA fragment coding for 139-amino-acid protein with MW 17 kDa, GenBank accession number AAK96887) were codon optimized for *E. coli* expression in pET28a expression vectors as a C-terminal His6 tagged proteins.^17^ Due to insoluble issue of the expressed rAra h 6-His proteins using pET28a, mature Ara 6.0101 (372 bp DNA sequences coding for 124-amino-acid protein, GenBank accession number AAD56337) was expressed as a N-terminal thioredoxin (TR)-Ara h 6.0101 fusion protein (MW 32 kDa) with a C-terminal His6 tag to facilitate purification using pET32a expression vector. The house dust mite allergen Der p 1-TR fusion protein was produced as a control. Gene synthesis and cloning services were provided by Bon Opus Biosciences.

### Recombinant peanut allergen production

The expression plasmid pET28a-Ara h 2.0201 and pET28a-Ara h 2.0101 were transformed into *E. coli* SHuffle strain (NEB C3026J)^18^ for production. The expression plasmid pET32a-Ara h 6-TR was transformed into *E coli* T7 strain (NEB C2527). A single colony of the transformed *E. coli* was cultured in 3mL 2YT media (10 g/L yeast extract, 16 g/L tryptone, 5 g/L NaCl), supplemented with 40 µg/mL kanamycin overnight at 30 °C with shaking at 250 rpm. These cultures (0.5mL) were used to inoculate 50mL (1:100 dilution) of 2YT medium and grown at 30 °C with shaking at 250 rpm until optical density (OD_600_) was 0.5 to 0.6 (about 4 h). The cultures were then separated into 3mL aliquots, each cultured in various concentrations of IPTG to induce protein expression overnight at varying temperatures (e.g. 16 °C, 25 °C, or 30 °C) with shaking at 250 rpm to define the optimal conditions for recombinant protein expression. The cell pellets were suspended in 1mL of lysis buffer (50 mM Tris, 10% glycerol, 0.1% Triton X-100) and sonicated using Dismemberator 705 (Fisherbrand) for 1 min (1%–3% intensity, 2-s pulses with 4-s pauses). After sonication, the samples were clarified by centrifugation for 20 min with 21,130 *g* at 4 °C. Each sample (5 µL) was loaded in a 10% or 12% Tris-glycine gel (Bio-Rad). Stain-free images were taken prior to standard WB procedure (Chemi Doc MP; Bio-Rad). Proteins were detected using anti-His Tag-HRP (1:500 [vol/vol]; BioLegend). The growth condition that yielded the most abundant protein was used for protein production.

### Purification of rAra h 2 and rAra h 6

Starter cultures of *E. coli* expression strains were used to inoculate 1 L of 2YT + 40 µg/mL kanamycin and grown following the previously determined optimal growth conditions for each recombinant protein. Bacteria were pelleted by centrifugation (4,347 *g*). Pellets in each 50mL tube left at −80°C for 30 min before resuspension in 20mL of lysis buffer. Each were sonicated on ice bath for 6 min of active sonication (20%–25% intensity, 2-s pulses with 10-s pauses). The lysates were then clarified by centrifugation using a high-speed centrifuge (20 min at 20,220 *g*; Thermo Scientific Sorval ST Plus Series Centrifuge) at 4 °C. The supernatants were adjusted to contain 25 mM imidazole (Thermo Fisher Scientific), 1 M NaCl, and 20 mM NaH_2_PO_4_. Then, 1mL of Ni-NTA Agarose (Marvelgent Biosciences) was added. After stirring for 1 h, the solution was added to a column (Thermo Fisher Scientific) and ran by gravity. The column was washed 10 times with wash buffer (20 mM imidazole, 1 M NaCl, pH 8.0). The bound protein was eluted with 3mL elution buffer (500 mM imidazole, 500 mM NaCl, 20 mM NaH_2_PO_4_, pH 7.4). The elution fraction was dialyzed overnight in 0.1X PBS with stirring. After dialysis, the solution was centrifuged for 10 min at 21130 *g* at 4 °C to remove precipitates. Protein concentration of the resulting supernatants was measured with NanoDrop One. The purity of rAra h 2.0201, rAra h 2.0101, and rAra h 6.0101 was estimated to be >80% using standard sodium dodecyl sulfate–polyacrylamide gel electrophoresis and Coomassie Blue stain.

### Affinity purification of Ara h 2 IgE from PNA samples

rAra h 2.0201 (1.5 mg/mL) was coupled to 0.3 g CNBr-activated Sepharose 4B (Cytiva) following manufacturer’s instruction. PNA and non-PNA serum (1mL) were diluted 1:1 with PBS and mixed with 0.4mL prepared Sepharose 4B media for 2 h at RT with agitation. After thoroughly washing the packed affinity column with >10 column volume of PBS, the binding antibodies were eluted with 1mL elution buffer (50 mM glycine, pH 2.75) that were immediately neutralized with 110 µL 1.5 M Tris-HCl, pH 8.8. The collected eluents were passed through a rProtein A agarose 4FF column (Marvelgent) to absorb Ara h 2–specific IgG. The flow-through fractions were dialyzed against 0.5 X PBS overnight, and the resulting fractions were examined for IgE level and used for downstream applications.

### Enzyme-linked immunosorbent assay

Indirect enzyme-linked immunosorbent assay (ELISA) was used to measure the binding capacity of the affinity-purified Ara h 2 IgE and the peanut allergen component IgE to various coated peanut allergen components. rAra h 2.0201, rAra h 1.0101, rAra h 6.0101-TR, rAra h 8, rAra h 9, and nAra h 3 (1 µg/mL) were coated to NUNC 96 well microtiter plate, half area (50 µL/well) in bicarbonate coating buffer (0.1 M, pH 9.6) at 4 °C overnight. The coated wells were washed 3 times with PBST (0.05% Tween 20) with automatic ELISA washing machine (BioTek 405 TS Microplate Washer), followed by blocking for 1 h with 10% FBS in PBST (75 µL/well) with shaking at 200 rpm. For affinity-purified Ara h 2 IgE binding test, the eluted IgE fractions were diluted to contain 1 ng/mL of total IgE using 10% FBS in PBST as a diluent. The diluted samples in duplicate were added into the blocked well (50 µL/well) for 1 h incubation at RT with shaking at 200 rpm. After washing 3 times, the goat anti-human IgE F(ab)2′-alkaline phosphatase conjugates (Kirkegaard & perry Laboratories, Gaithersburg, MD)(1:3000 dilution) was added (50 µL/well) for 1 h incubation at RT. Following washing the plate 6 times with PBST, the freshly made *p*-nitrophenyl phosphate substrate solutions were added for color development, and the outcomes were read with Varioskan LUX (Thermo Fisher Scientific) at 405 nM wavelength. For the Ara h 2 binding test, Ara h 2 IgE E-11F10^16^ was used as a reference standard, and the binding value was expressed as IU/mL. For IgE binding to rAra h 1 and rAra h 6-TR, the binding strengths were expressed as OD_405_ due to lack of the corresponding IgE standards at the time of experiments were performed. An in-house expressed Der p 1-TR (1 µg/mL) as a coat for negative control. The OD_405_ value of negative control was <0.02. The total IgE level eluted from the Ara h 2 affinity chromatography was determined by a previously described sandwich ELISA.^19^

### Passive cutaneous anaphylaxis

Passive cutaneous anaphalaxis (PCA) to determine peanut allergic IgE-mediated allergic reactions triggered by peanut allergen components was performed with a protocol established previously.^20–24^ The dorsal skin of human high affinity IgE receptor 1 alpha chain (hFcεRIα) transgenic mouse^25–27^ was shaved, and the injection sites were marked with red Sharpie pen. Fifty microliters (50 µL) of 1:4 diluted PNA serum, the affinity-purified IgE fraction, the defined concentration of Ara h 1 IgE and Ara h2 IgE, and the mixture of them was intradermally injected to each spot for IgE sensitization. Forty-eight hours later, CPE (10 µg/mouse) or defined amount of the peanut allergen component, mixed with 1% Evans blue dye (100 µL/mouse), was intravenously injected via tail vein. The PCA positive control site was injected with the anti-FcεRIα mAb clone AER-37 at 0.3 µg/mL 10 min before allergen challenge to confirm the functionality of mast cell degranulation. A blue spot indicative of allergic reactions would appear within 15 min in the serum sensitization site(s) if allergic reactions were triggered. Mice were euthanized 30 min postchallenge. The dorsal skin was removed and reversed for photographing to assess local allergic reactions. Using hFcεRIα transgenic mice for this study was approved by local Institutional Animal Care and Use Committee animal research committee (project 22871-01).

## Results

### Peanut protein–interacting IgE patterns in a soy protein blocking WB

Eight clinically confirmed PNA serum samples and 1 peanut IgE sensitized but not allergic serum sample as a control were used throughout the study. Demographic characteristics, including peanut IgE and the available peanut component IgE data, are presented in Table 1.

**Figure 1.**
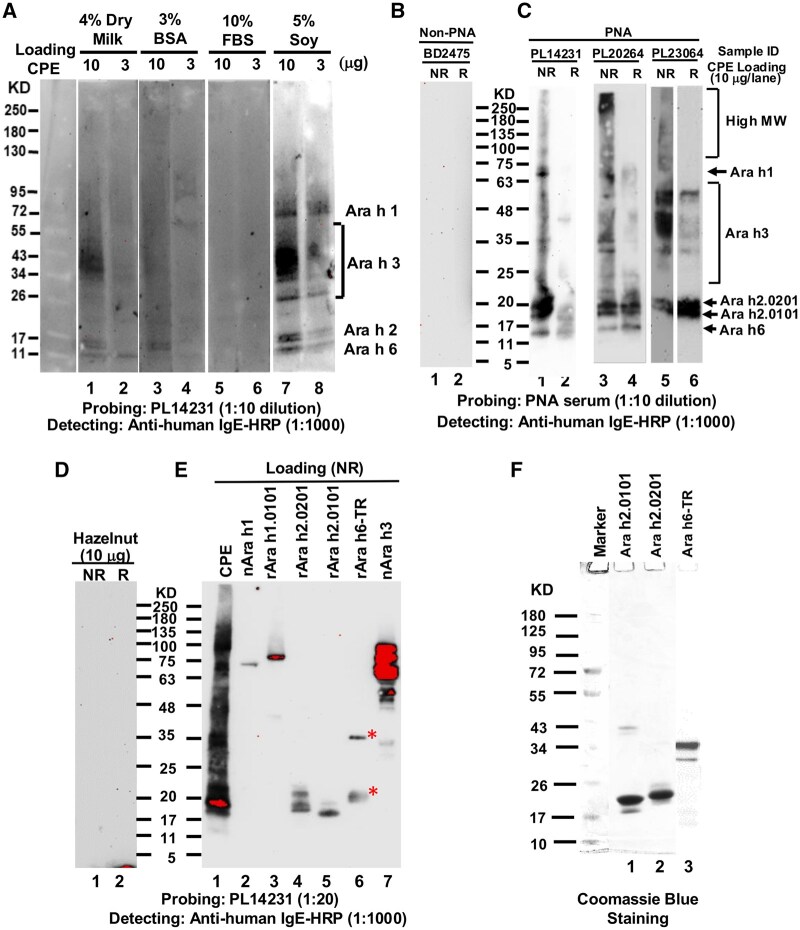
Analysis of peanut allergen binding IgE using a modified WB with soy proteins as blocking solution. (A) Comparison of blocking effects of various blocking reagents for peanut allergic IgE detection. (B) IgE patterns of a non-PNA sample BD2475. (C) IgE patterns of 3 PNA samples (PL14231, PL20264, PL23064). IgE signals corresponding to high MW, Ara h 1, Ara h 2.0201 and Ara h 2.0101 isoforms, and Ara h 6 were annotated. (D) IgE patterns probing with PNA sample PL14231 in hazelnut fractionated blot. (E) IgE binding patterns of peanut allergen components. A part of the rAra h 6-TR was broken down to ∼20 kDa fragments, leading to 2 IgE binding bands (annotated by the red asterisks). (F) Purification of the in-house produced recombinant peanut allergens used in this study. Dilutions of sample and anti-human IgE-HRP were indicated under the corresponding blots.

Although WB has been an indispensable tool identifying allergen interactive IgE involving allergic reactions, the peanut allergen binding IgE using PNA serum frequently resulted in high background binding using the traditional WB blocking protocol, resulting in unsatisfied IgE signals including indistinguishable Ara h 2 isoforms (Fig. 1A, lanes 1–6). Among several blocking reagents tested (4% dry milk, 3% bovine serum albumin, 10% FBS, and 5% soy protein solution), a previously investigated soy protein as a blocking reagent^28^ appeared working better than other traditional WB blocking (Fig. 1A). The refined protocol using soy solution containing 10 mg/mL soy protein could not only reduce nonspecific binding background, but also allow reproducible detection of IgE signal from PNA serum, including IgE binding to 2 Ara h 2 isoforms, Ara h 1, Ara h 6, and Ara h 3 (Fig. 1C). Such IgE signal patterns were not seen using non-PNA serum BD2475 (Fig. 1B). The protocol only permitted detection of IgE signals bound to fractionated peanut protein but not to hazelnut proteins probed with PNA serum (Fig. 1D). In peanut allergen component fractionated blot, IgE bound to each component could be clearly detected (Fig. 1E, lanes 2–7). The concerns of the potential peanut allergen cross-reactive component in soy proteins^29^ that block the peanut allergic IgE detection appeared not relevant as IgE signals, particularly to the two Ara h 2 isoforms, could be clearly detected in all the PNA samples but not the non-PNA samples. These data collectively demonstrated that IgE of PNA against the previously characterized major peanut allergens could be specifically and reproducibly detected with this modified WB.

**Figure 2.**
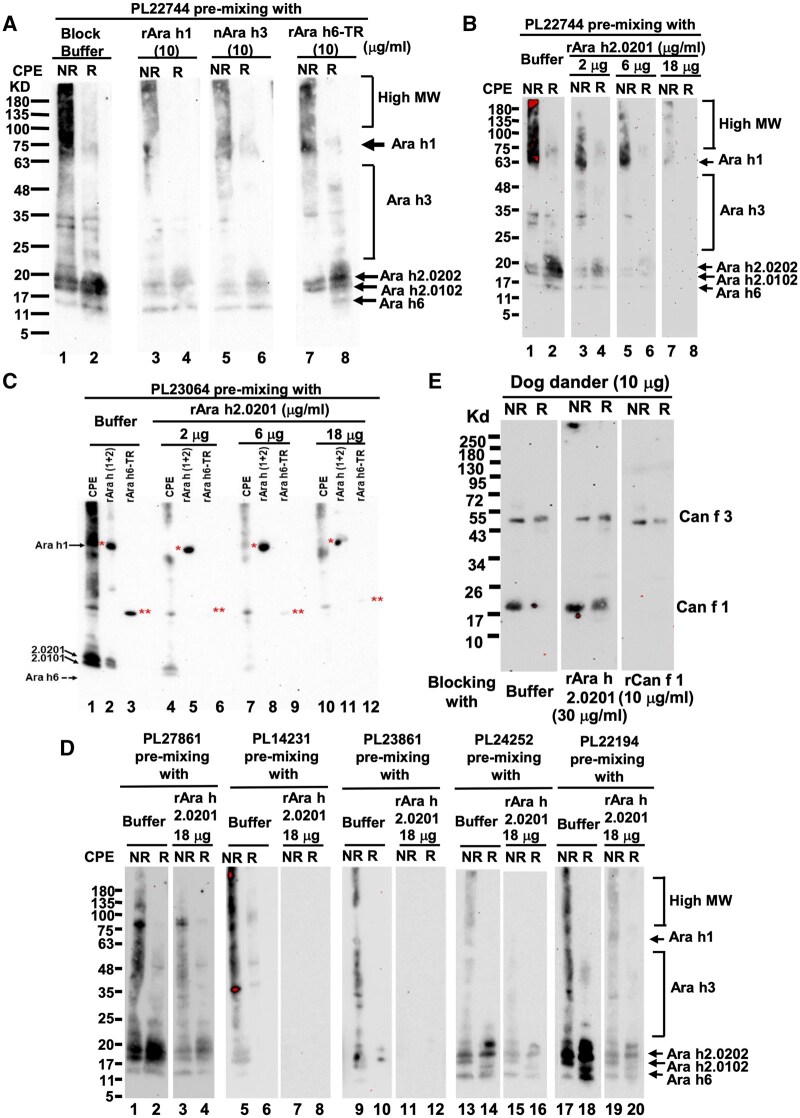
Cross-inhibition of peanut allergen binding IgE in WB. (A) Inhibition patterns of the peanut protein binding IgE by rAra h 1, nAra h 3, and rAra h 6-TR. (B) Inhibition pattern of the peanut protein binding IgE of PNA sample PL22744 by various concentration of rAra h 2.0201. (C) Inhibition patterns of the peanut protein binding IgE of PNA sample PL23064 by various concentration of rAra h 2.0201. One red asterisk annotated the positions of Ara h 1 and 2 red asterisks annotated the positions of rAra h 6-TR. Although signal bound to nAra h 6 of CPE was not seen under the low-exposure conditions, it could be seen with high exposures (data not shown). (D) Inhibition patterns of the peanut protein binding IgE of PNA samples by rAra h 2.0201 at 18 µg/mL. (E) rAra h 2.0201 did not block the dog allergic IgE binding to Can f 1 and Can f 3 in WB. All the WB conditions were the same as for those presented in Fig. 1, except that the samples were diluted 1:10.

Under NR conditions, PNA samples always displayed smeared IgE signal patterns throughout the entire fractionated peanut protein spectrum covering the ranges from ∼10 kDa to larger than 180 kDa, including the molecular weight higher than that of Ara h 1 (hereafter collectively referred as high MW), the MW size ranges corresponding to Ara h 1, Ara h 3, Ara h 2 (both Ara h 2.0201 and Ara h 2.0101 isoforms), and Ara h 6 (Fig. 1C, E). IgE signal patterns, including intensities of the smeared signals and relative positions of IgE-interactive bands, appeared unique to each PNA sample, as no consistent common patterns, except for the 2 Ara h 2 isoforms, were observed among PNA samples tested (Fig. 1C, E; also see Fig. 2).

Under R conditions, the smeared IgE signals, particularly high MW, bound to the broad ranges of fractionated peanut proteins seen under NR conditions either disappeared or significantly weakened (Fig. 1C), indicating that the smeared IgE signals under NR conditions did not represent nonspecific binding. The smeared IgE signal patterns were only seen with fractionated CPE but not with the purified or recombinant component, further supporting this notion (Fig. 1E, lane 1 vs lanes 2–7). IgE signal bound to Ara h 2 and/or Ara h 6 could be either decreased (PL14231, PL20264 of Fig. 1C; PL23861 in Fig. 2D), or increased (PL23064 in Fig. 1C; PL22744 in Fig. 2A, B; PL27861, PL 24252, and PL22194 in Fig. 2D) under R compared with NR conditions (Figs. 1C and 2). Notably, the IgE binding bands corresponding to Ara h 1 position were mostly eliminated, or significantly reduced, under R conditions (Figs. 1C, 2A, 2B, and 2D).

### Cross-inhibition of PNA IgE signals by peanut allergens

Previous studies suggested that there were variable levels of cross-reactivity among peanut allergen–specific IgE, as one given type of peanut allergen binding IgE could be cross-inhibited by other type(s) of peanut allergens.^9–13^ To better understand the basis of this cross-reactivity, we investigated IgE cross-inhibition using WB with same experimental conditions to facilitate IgE signal intensity comparison within the same panel. Both purchased or in-house produced recombinant peanut allergen components (Fig. 1F), except for Ara h 3, were used for inhibition due to potential contamination concerns of the purified components from natural peanut proteins.^15^

As shown in Fig. 2A, PL22744, without cross-inhibition by peanut allergen component, displayed an IgE binding pattern like that of the PNA samples presented in Fig. 1C. rAra h 1.0101 inhibited IgE binding to Ara h 1, as well as slightly reduced IgE bound to the overall fractionated peanut proteins. nAra h 3 produced a similar inhibition pattern comparable to that of rAra h 1.0101. rAra h 6-TR inhibited IgE binding to Ara h 6, as well as slightly reduced IgE signals bound to overall peanut protein smears under NR conditions. These data collectively showed that rAra h 1, nAra h 3, and rAra h 6-TR could inhibit IgE binding to their corresponding allergens, as well as mildly reduced IgE binding to the entire fractionated peanut protein spectrum.

When rAra h 2.0201 was assessed for inhibition test, not only did Ara h 2 (both Ara h 2.0201 and Ara h 2.0101 isoforms) bind IgE, but also the IgE bound to the broad peanut protein spectrum, including the areas of high WM, Ara h 1, Ara h 3, and Ara h 6, were profoundly inhibited, in a rAra h 2 dose-dependent fashion (Fig. 2B).

To test whether rAra h 2 also inhibited IgE binding to recombinant peanut allergen components, CPE (lane 1), rAra h 1.0101, an rAra h 2.0201 and rAra h 2.0101 mixture as a single loading sample (lane 2), and rAra h 6.0101-TR (lane 3) (Fig. 2C) were fractionated for WB. Without rAra h 2.0201 inhibition, PL23064 IgE signals bound to the fractionated peanut proteins as a smear (lane 1), rAra h 1.0101 (lane 2, indicated with 1 red asterisk), rAra h 2.0201 and rAra h 2.0101 (lane 2), and rAra h 6-TR (lane 3, indicated with double red asterisks), were apparent. Inhibition using rAra h 2.0201 demonstrated that it could profoundly inhibit not only IgE binding to both natural and recombinant Ara h 2 (for both isoforms), but also rAra h 6-TR IgE binding, in a dose-dependent manner (Fig. 2C). In addition, rAra h 2.0201 also strongly inhibited IgE binding to nAra h 1 from CEP (Fig. 2C, lane 1 vs lanes 4, 7, and 10), but less effective to rAra h 1.0101, as higher concentration was required to achieve partial inhibition (Fig. 2C, lane 2 vs lanes 5, 8, and 11).

Among other PNA samples tested, rAra h 2.0201 displayed inhibiting effects on the IgE signals bound to all peanut proteins, either almost completely (for PL14231 and PL23861) or partially (for PL27861, PL24252 and PL22914) (Fig. 2D), particularly inhibiting the IgE species bound to the MW ranges higher than that of Ara h 2. Taken together, these WB results revealed that Ara h 2–specific IgE, in addition to bind to Ara h 2 itself, were also able to cross-react with a large swath of peanut proteins including Ara h 1, Ara h 3, and Ara h 6.

In some samples, such as PL24252 and PL22914, there were IgE binding bands lower than that of the presumable Ara h 6 position (Fig. 2D), which likely corresponded to the presumable Ara h 8 and/or Ara h 9, but their nature was not further investigated. These IgE binding bands were also partially inhibited by rAra h 2.0201, indicating that Ara h 2 IgE was also responsible for the cross-reactivity with these smaller peanut proteins. WB results of peanut IgE cross-inhibition presented in Fig. 2 are summarized in Tables 2 and 3.

**Table 2.** Inhibition of IgE bound to CPE by peanut allergen components.

		PL22744	PL23064	PL27861	PL14231	PL23861	PL24252	PL22914

Allergen	Component (µg/mL)	NR R	NR R	NR R	NR R	NR R	NR R	NR R
**CPE**	**Blocking buffer**	**− −**	**− −**	**− −**	**− −**	**− −**	**− −**	**− −**
**CPE**	**rAra h 1.0101 (10)**	**+ +**						
**CPE**	**nAra h 3 (10)**	**+ +**						
**CPE**	**rAra h 6.0101 (10)**	**+ +**						
**CPE**	**rAra h 2.0201 (2)**	**+ +**	**++**					
**CPE**	**rAra h 2.0201 (6)**	**++ ++**	**++**					
**CPE**	**rAra h 2.0201 (18)**	**+++ +++**	**++**	**+ +**	**++++ ++++**	**++++ ++++**	**++ ++**	**++ ++**

**Table 3.** Inhibition of IgE bound to peanut allergen components by rAra h 2.

Peanut allergen component	rAra h 2.0201			
	0 (Buffer)	2 µg/mL	6 µg/mL	18 µg/mL
**rAra h 1.0101**	**−**	**−**	**−**	**−**
**rAra h 2.0101**	**−**	**+**	**+++**	**++++**
**rAra h 2.0201**	**−**	**+**	**+++**	**++++**
**rAra h 6.0101-TR**	**−**	**++++**	**+++**	**+++**

To demonstrate that rAra h 2–mediated broad inhibition of IgE cross-reactivity among multiple peanut allergen components is specific, rAra h 2.0201 was used to inhibit dog allergic IgE binding in WB. As shown in Fig. 2E, rAra h 2.0201 up to 30 µg/mL did not exhibit inhibitory effects on both *Canis familiaris* 1 (Can f 1) and Can f 3 IgE binding, whereas Can f 1 IgE binding, but not Can f 3 IgE binding, was specifically blocked by the recombinant Can f 1 at 10 µg/mL as a positive control for the test.

### Affinity purified Ara h 2 IgE reconstituted the broad IgE binding patterns to peanut proteins

Inhibition of IgE binding to the broad peanut protein spectrum by rAra h 2 suggests that Ara h 2–specific IgE cross-reacts with multiple peanut proteins. To test whether Ara h2-specific IgE was directly responsible for IgE cross-binding to the peanut proteins/allergens in addition to Ara h 2 itself, the Ara h 2–specific IgE was affinity-purified from PNA, with a non-PNA sample as a control, and subsequently used to probe the blots containing fractionated peanut proteins from CPE.

**Figure 3.**
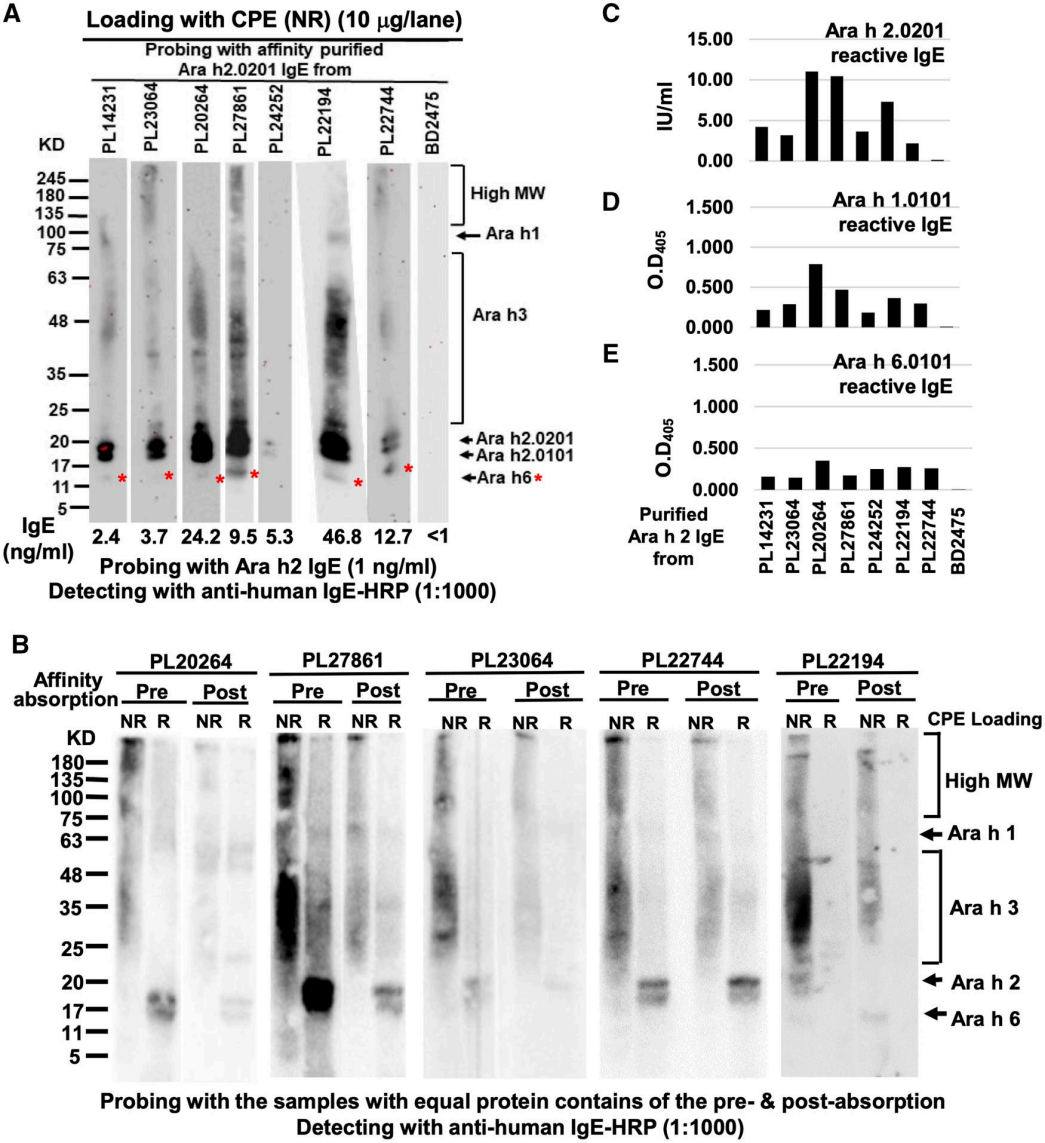
Ara h 2 IgE-mediated peanut protein binding patterns. (A) Binding patterns of the affinity-purified Ara h 2 IgE to the fractionated peanut proteins in WB. IgE levels of the affinity-purified IgE of each sample were presented in the bottom of each corresponding WB strips, and the smeared IgE signals corresponding to peanut proteins/allergens were annotated on the left, with IgE signals corresponding to Ara h 6 indicated with a red asterisk. (B) Reduction of IgE binding signals from the post–rAra h2.0201 affinity chromatography absorbed flow-through fractions compared with that of the preabsorbed serum. The loading protein contents were adjusted to be equal for pre- and postabsorption for each sample. In the ELISA, the affinity-purified Ara h 2 IgE bound to rAra h 2.0201 (C), rAra h 1.0101 (D), and rAra h 6.0101-TR (E). OD_405_ for blank was <0.02.

Seven (with 1 sample lost during preparation therefore not tested) out of 8 PNA samples, but not the non-PNA samples, eluted from Ara h 2.0201 affinity chromatography contained detectable IgE (Fig. 3A, bottom). These Ara h 2 affinity–purified IgE, diluted to 1 ng/mL, most prominently bound to Ara h 2 (both Ara h 2.0201 and Ara h 2.0101 isoforms) in WB, indicating that these eluted IgE from the affinity chromatography preserved Ara h 2 binding activity (Fig. 3A). These Ara h 2–specific IgE displayed variably smeared IgE binding patterns, with IgE signals bound to the areas of high MW, Ara h 1, Ara h 3, and Ara h 6, except for PL24252, due to minimum level of Ara h 2 IgE presented (Fig. 3A, lane 5), whereas the eluted fraction of the non-PNA sample failed to produce detectable IgE signal (Fig. 3A, right-most lane). These data directly demonstrated that affinity-purified Ara h 2–specific IgE was able to bind to the broad peanut protein species in addition to Ara h 2 itself.

We also examined IgE signal patterns of PNA serum post–affinity absorption, testing the assumption that IgE signal levels binding to broad peanut proteins, in addition to Ara h 2, would corroboratively decrease along with the Ara h 2 IgE signal level if Ara h 2 IgE was responsible for the cross-reactivity among multiple peanut proteins. The results from 5 samples showed that the flow-through fractions from the postaffinity chromatography absorption indeed displayed similar patterns of lower Ara h 2–specific IgE as well as lower IgE cross-reactivity with broad peanut proteins/allergens compared with the preabsorbed samples (Fig. 3B). These data indirectly support that Ara h 2–specific IgE was associated with IgE signals bound to the broad peanut proteins in addition to that of Ara h 2.

To further test whether the affinity-purified Ara h 2 IgE was able to bind to other peanut allergen components, rAra h 1 and rAra h 6-TR, alone with rAra h 2.0201 as a positive control, were used as coat at 1 µg/mL in indirect ELISA. The affinity-purified IgE fractions were diluted to contain 1 ng/mL of IgE level for measurement. All the affinity-purified Ara h 2 IgE from 7 PNA samples, but not from the non-PNA sample, was able to bind to Ara h 2.0201 (Fig. 3C) as well as to rAra h 1 (Fig. 3D) and rAra h 6-TR (Fig. 3E), confirming their ability to bind to Ara h 1 and Ara h 6. As a negative assay control, a recombinant Der p 1-TR coated wells did not yield signal higher than that of blocking buffer as assay blank (OD_405_ < 0.020, data not shown).

### Cross-reactivity of monoclonal human IgE against peanut allergen component

**Figure 4.**
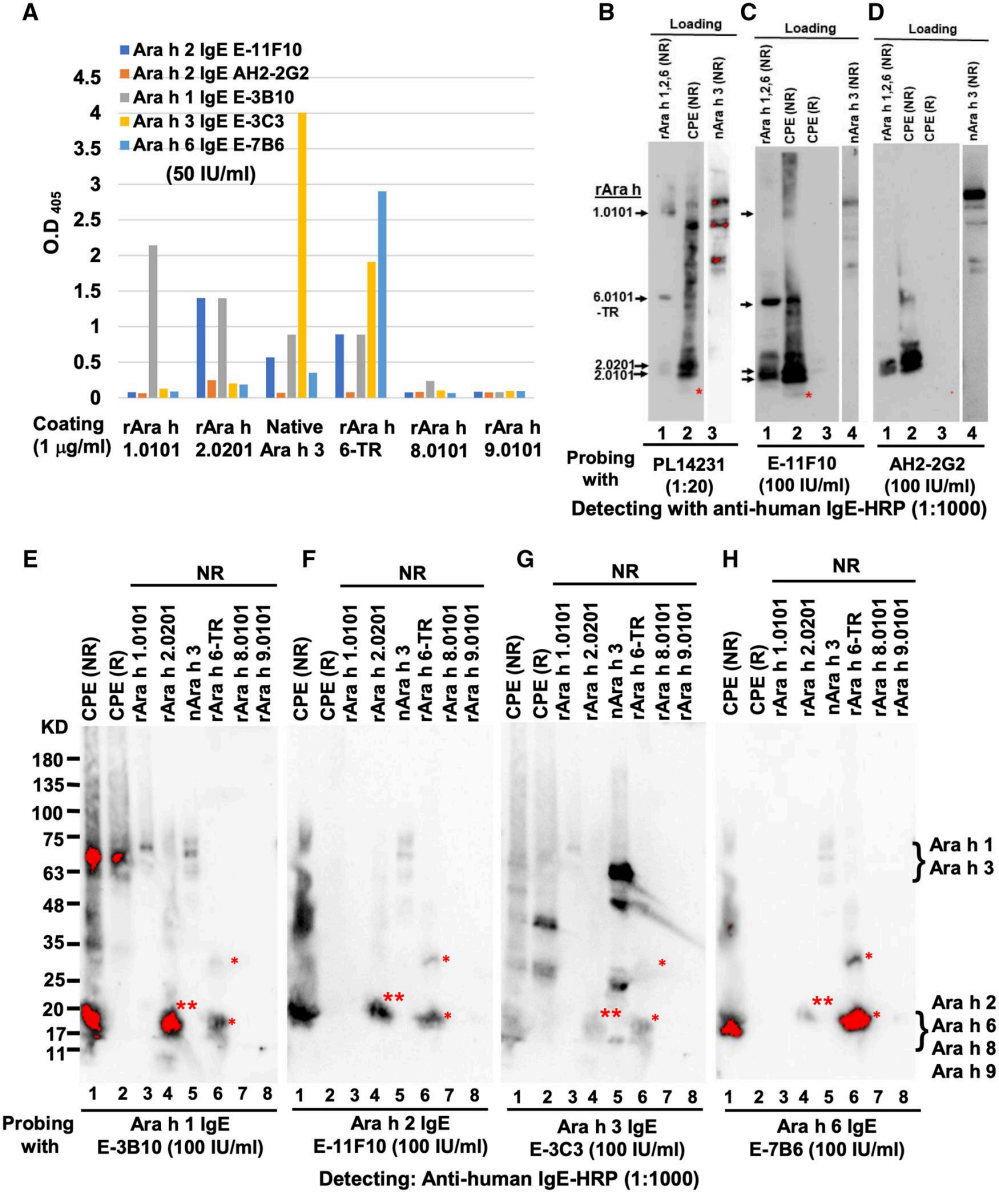
Binding profiles and patterns of human IgE against peanut component. (A) Binding profiles of IgE to various peanut allergen components in ELISA. (B) Binding of PNA PL14231 IgE to the peanut allergen components in WB. The red asterisk annotated the binding of PNA IgE to nAra h 6. (C, F) Binding patterns of Ara h 2 human IgE E-11F10 in WB. (D) Binding patterns of Ara h 2 chimeric IgE AH2-2G2 in WB. (E) Binding patterns of Ara h 1 IgE E-3B10 in WB. (G) Binding patterns of Ara h 3 IgE E-3C3 in WB. (H) Binding patterns of Ara h 6 IgE E-7B6 in WB. This blot was stripped and reprobed with Ara h 2 IgE E11F10 to produce the blotting results shown in panel F. The single red asterisk annotated the IgE signal corresponding to the positions of nAra h 6, rAra h 6-TR, and its breakdown fragment, whereas double red asterisks annotated the binding position of Ara h 2 IgE.

We further examined peanut allergen binding profiles of monoclonal human IgE using ELISA. IgE binding profiles revealed that at 50 IU/mL level, the human Ara h 2 IgE E-11F10 apparently bound to rAra h 2, nAra h 3, and rAra h 6; Ara h 1 IgE E-3B10 strongly bound to rAra h 1, rAra h 2, nAra h 3, and rAra h 6, and weakly to rAra h 8; Ara h 3 IgE E-3C3 strongly bound to nAra h 3 and rAra h 6, and weakly to rAra h 2; Ara h 6 IgE E-7B6 strongly bound to rAra h 6 and weakly to rAra h 2 and nAra h 3 (Fig. 4A).

To analyze Ara h 2 IgE binding patterns in WB, rAra h 1, rAra h 6-TR, rAra h 2.0201, and rAra h 2.0101 were combined as a single sample loaded beside fractionated CPE for comparison, and with nAra h 3 loaded in a separate lane for Ara h 2 IgE binding test. As a reference, IgE of PNA PL14231 bound to a swath of peanut proteins from CPE as smeared signals, and to each peanut allergen component in the combined loading lane, as well as to nAra h 3 (Fig. 4B). In addition to binding to 2 Ara h 2 isoforms, Ara h 2 IgE E-11F10 bound to the smeared high MW peanut proteins from CPE, nAra h 1 from CPE, nAra h 3, and Ara h 6 from both CPE and rAra h 6 (Fig. 4C), whereas another Ara h 2 IgE AH2-2G2, a chimeric mouse-human IgE derived from a human IgE Fc knock-in mouse immunized with Ara h 2 (www.allermabs.com), bound to Ara h 3 from both CPE and purified nAra h 3 (Fig. 4D, lanes 1 and 3).

In WB, Ara h 1 IgE E-3B10 bound to the NR and R Ara h 1 from CPE, and NR rAra h 1, rAra h 2, nAra h 3, and rAra h 6 (Fig. 4E); Ara h 2 IgE E-11F10 bound to the NR Ara h 2 and Ara h 3 from CPE, and NR rAra h 2, nAra h 3, and rAra h 6 (Fig. 4F); Ara h 3 IgE E-3C3 bound to multiple proteins from CPE under both R and NR conditions, and to NR rAra h 1, rAra h 2, nAra h 3, and rAra h 6 (Fig. 4G); Ara h 6 IgE E-7B6 bound to NR Ara h 2 and Ara h 3 from CPE, rAra h 2, nAra h 3, and rAra h 6, and weakly to rAra h 9 (Fig. 4H). A common feature of these results revealed the Ara h 1, Ara h, Ara h 3, and Ara h 6 human IgE could broadly interact with other peanut allergen components, and all of them could bind to Ara h 2/Ara h 6 via a conformational epitope that could be eliminated under reducing conditions.

### Cross-reactivity of mouse anti-peanut allergen component mAb

**Figure 5.**
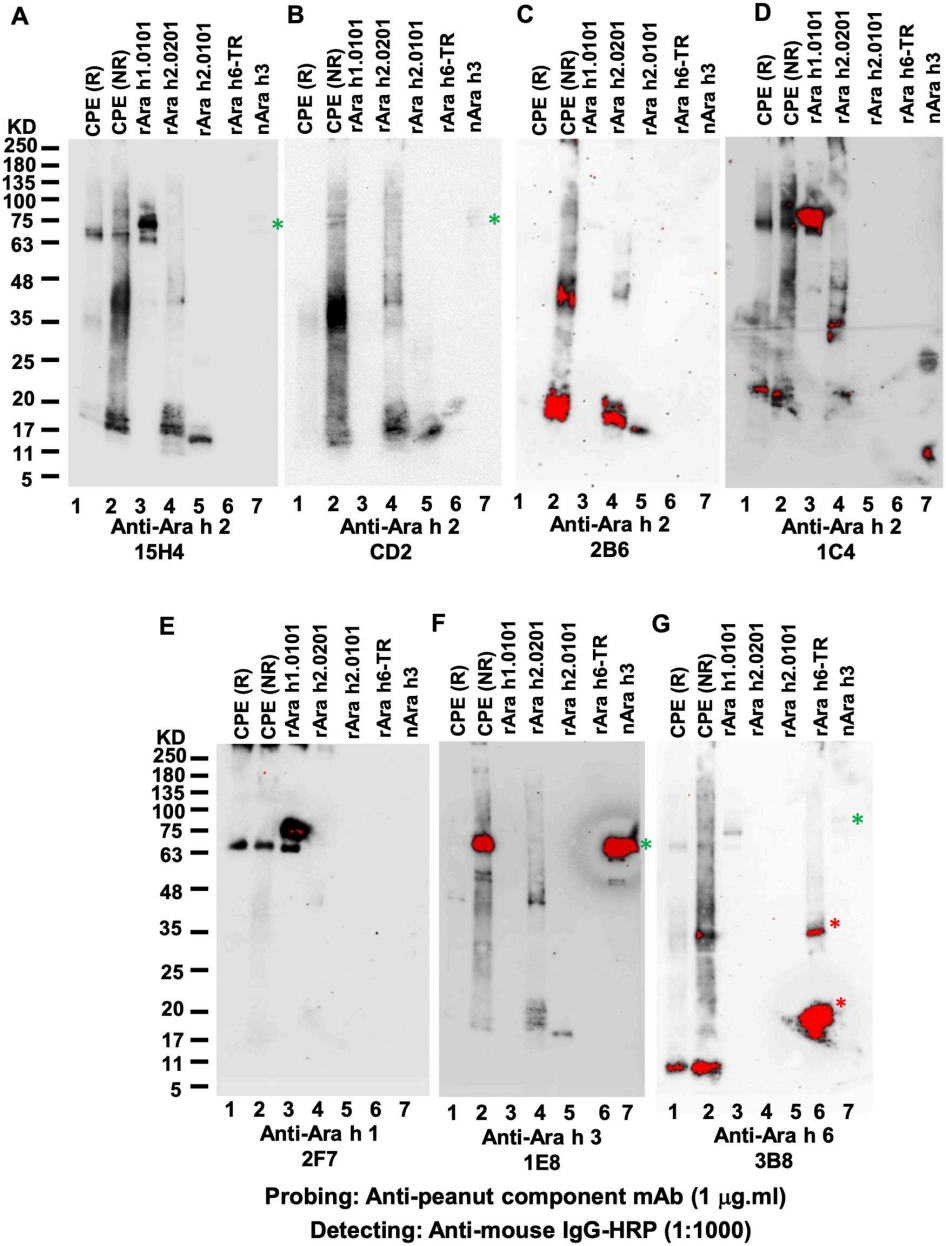
Binding profiles and patterns of mouse mAb anti-peanut allergen components. (A) Anti-Ara h 2 clone 15H4. (B) Anti-Ara h 2 clone CD2. (C) Anti-Ara h 2 clone 2B6. (D) Anti-Ara h 2 clone 1C4. (E) Anti-Ara h 1 clone 2F7. (F) Anti-Ara h 3 clone.1E8. (G) Anti-Ara h 6 clone 3B8. The green asterisks annotated Ara h 3 IgE binding signals, whereas red asterisks annotated Ara h 6-TR and its breakdown fragment.

We further surveyed the cross-reactivity of a series of randomly picked mouse anti-peanut allergen components using WB. Among 4 anti-Ara h 2 mAb clones tested, 15H4 could bind not only to R and NR Ara h 2, but also to Ara h 1 and Ara h 3 (Fig. 5A), whereas CD2 bound to Ara h 2 as well as to Ara h 6 and Ara h 3 but not to Ara h 1 (Fig. 5B). Clone 2B6 could only bind to NR but not R Ara h 2 (Fig. 5C). Clone 1C4 only displayed weak Ara h 2 binding but stronger Ara h 1 binding, and moderate Ara h 3 binding (Fig. 5D). The anti-Ara h 1 clone 2F7 was bound to both R and NR nAra h 1 (Fig. 5E). Anti-Ara h 3 clone 1E8 bound to NR but not to R Ara h 3, and to Ara h 2 (Fig. 5F). Anti-Ara h 6 clone 3B8 could not only bind to both NR and R Ara h 6, but also to Ara h 1 and Ara h 3 (Fig. 5G). The binding patterns of these anti-peanut allergen component mAbs, including human IgE and mouse IgG mAb, were summarized in Table 4.

**Table 4.** Summary of the binding capacity of the anti-penaut allergen component mAb.

Clone	Target	Ig Type	Binding to						Assay	Source
			Ara h 1	Ara h 2	Ara h 3	Ara h 6	Ara h 8	Ara h 9		
**E-11F10**	**Ara h 2**	**Human IgE**	**No**	**Yes**	**Yes**	**Yes**	**No**	**No**	**WB, ELISA**	**InBio**
**AH2-2G2**	**Ara h 2**	**Chimeric IgE**	**No**	**Yes**	**Yes**	**No**	**No**	**No**	**WB**	**AllerMabs**
**E-3B10**	**Ara h 1**	**Human IgE**	**Yes**	**Yes**	**Yes**	**Yes**	**Yes**	**No**	**WB, ELISA**	**InBio**
**E-3C3**	**Ara h 3**	**Human IgE**	**No**	**Yes**	**Yes**	**Yes**	**No**	**No**	**WB, ELISA**	**InBio**
**E-7B6**	**Ara h 6**	**Human IgE**	**No**	**Yes**	**Yes**	**Yes**	**No**	**No**	**WB, ELISA**	**InBio**
**15H4**	**Ara h 2**	**Mouse IgG**	**Yes**	**Yes**	**Yes**	**No**	**ND**	**ND**	**WB**	**Allerdia**
**CD2**	**Ara h 2**	**Mouse IgG**	**Yes**	**Yes**	**Yes**	**No**	**ND**	**ND**	**WB**	**Allerdia**
**2B6**	**Ara h 2**	**Mouse IgG**	**Yes**	**Yes**	**No**	**No**	**ND**	**ND**	**WB**	**InBio**
**1C4**	**Ara h 2**	**Mouse IgG**	**Yes**	**Yes**	**Yes**	**No**	**ND**	**ND**	**WB**	**InBio**
**2F7**	**Arah 1**	**Mouse IgG**	**Yes**	**Yes**	**No**	**No**	**ND**	**ND**	**WB**	**InBio**
**E8**	**Ara h 3**	**Mouse IgG**	**No**	**Yes**	**Yes**	**No**	**ND**	**ND**	**WB**	**InBio**
**3B8**	**Ara h 6**	**Mouse IgG**	**Yes**	**Yes**	**Yes**	**Yes**	**ND**	**ND**	**WB**	**InBio**

ND, not determined.

### Potency of peanut allergen component in triggering allergic reactions in vivo

Ara h 2 has been firmly established as the most potent peanut allergen component triggering PNA; however, the role and relative contributions of Ara h 1, Ara h 3, and Ara h 6 in PNA are not well demonstrated, although IgE of PNA binds to these components. We determined the potencies of peanut allergen components in triggering peanut allergic reactions using a PCA assay^19–24^ with a hFc εRIα transgenic mouse model.^25–27^

**Figure 6.**
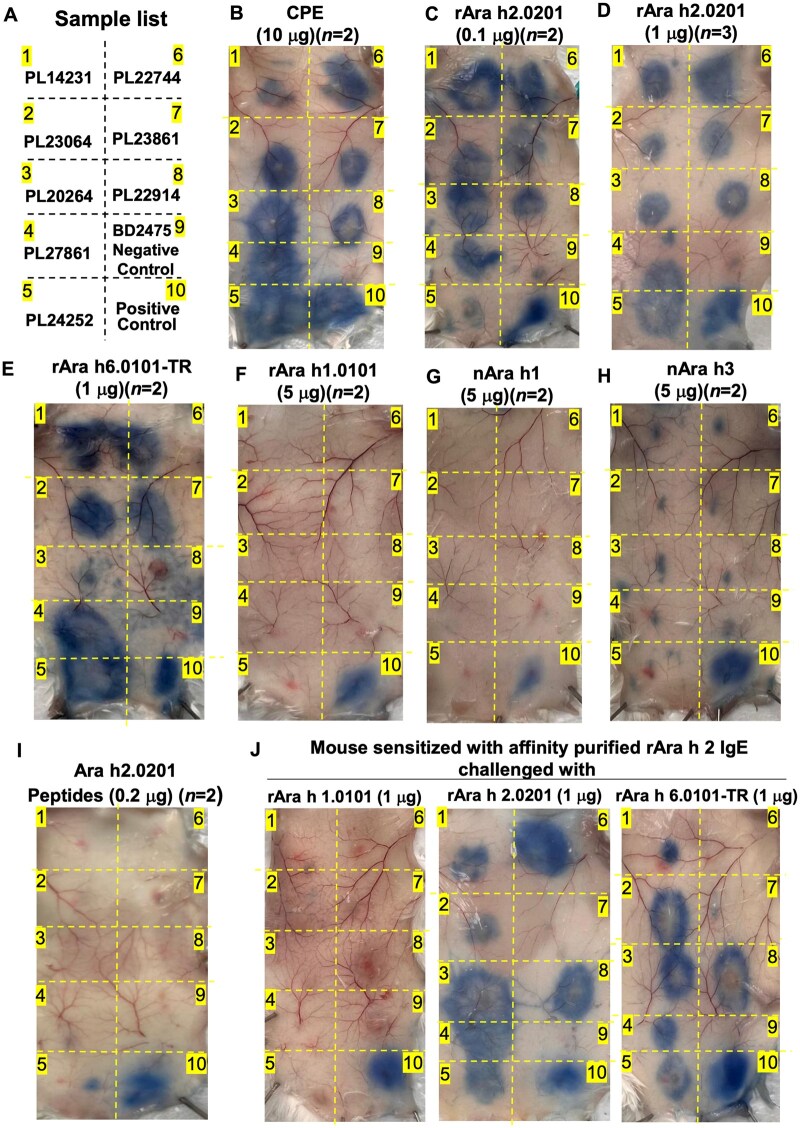
Potency of peanut allergen components in triggering peanut allergic reactions in PCA. (A) Diagram of sample injection positions. Spots 1 to 8 were sensitized with PNA samples, spot 9 was non-PNA sample BD2475, and spot 10 was the PCA positive control. PCA outcomes challenged with (B) CPE (10 µg); (C) rAra h 2.0201 (0.1 µg); (D) rAra h 2.0201 (1 µg); (E) rAra h 6.0101-TR (1 µg); (F) rAra h 1.0101 (5 µg); (G) nAra h 1 (5 µg); (H) nAra h 3 (5 µg); (I) Ara h 2.0201 peptides containing 3 repeated DPYSPS motifs (0.2 µg); and (J) Ara h 2 affinity-purified IgE sensitized animals challenged with rAra h 1.0101 (1 µg), rAra h 2.0201 (1 µg), and rAra h 6.0101-TR (1 µg), respectively.

Sample sensitization positions are diagramed in Fig. 6A. Robust allergic reactions occurred at all 8 PNA sample injection sites (sites 1–8) but not at the non-PNA sample injection site (site 9), when challenged with CPE (Fig. 6B), validating that these PNA samples contained allergic IgE capable of mediating peanut allergic reactions upon peanut allergen challenge.

Individual peanut allergen components were used to challenge the animals sensitized with PNA samples to determine its potential triggering capacity of peanut allergic reactions. rAra h 2.0201 at 0.1 µg (5.2 pM) (Fig. 6C) and 1 µg (52 pM) (Fig. 6D), respectively, triggered a similar level of PCA reaction comparable to that by 10 µg CPE (Fig. 6B), indicating that rAra h 2.0201 at the 0.1 µg level already could trigger robust peanut allergic reactions. rAra h2.0101 at 1 µg also triggered PCA reactions comparable with that of rAra h 2.0201 (data not shown). rAra h 6.0101-TR at 1 µg (31 pM) triggered apparent PCA reactions in 6 of 8 samples (Fig. 6E), with the remaining 2 of 8 samples (sites 3 and 8) displaying weak activity.

rAra h 1 and nAra h 1 at 1 µg (15.6 pM) or 5 µg (78 pM) level, and nAra h 3, either at the 1 µg or 5 µg level (pM level not calculated due to multiple protein species presented) (Fig. 1E, lane 7), failed to trigger PCA reactions (Fig. 6F–H), indicating that Ara h 1 and/or Ara h 3 were not able to trigger peanut allergic reactions at concentration ∼15-fold higher (molar amount) than that of rAra h 2.0201 with robust PCA triggering capacity (Fig. 6C).

Ara h 2.0201 peptide ^42^DPYSPSQDPYSPSQDPDRRDPYSPS^66^ in the hypervariable loop region contained 3 repeated DPYSPS motifs (underlined) that were thought important for Ara h 2’s allergenicity.^30–32^ Challenging PNA serum–sensitized animals with this peptide did not result in allergic reactions seen with rAra h 2.0201 in all 8 PNA samples (Fig. 6I), indicating that the linear DPYSPS motif was not sufficient to trigger peanut allergic reactions.

Ara h 2 affinity-purified, IgE-sensitized animals were responsive to rAra h 2.0201 and rAra h 6.0101-TR challenge, indicating that peanut allergic reactions mediated by Ara h2 IgE was triggered not only by Ara h 2, but also by Ara h 6 (Fig. 6J). However, rAra h 1.0101 challenge was not able to trigger Ara h 2–specific IgE-mediated allergic reactions (Fig. 6J), indicating that rAra h 1.0101 was not able to trigger Ara h2 IgE-mediated peanut allergic reactions, even though Ara h 2 IgE could bind to Ara h 1. The peanut component challenge results are summarized in Table 5.

**Table 5.** Summary of PCA results.

PCA			1	2	3	4	5	6	7	8	9	10
Panel	Sensitization	Challenge (µg)	PL14231	PL23064	PL20264	PL27861	PL24252	PL22744	PL23861	PL22914	BD2475	PCA (+) Ctrl
**B**	**Serum**	**CPE (**10**)**	**++**	**+++**	**+++**	**+++**	**+++**	**+++**	**+++**	**+++**	−	**+**
**C**	**Serum**	**rAra h 2.0201 (0.1)**	**++**	**+++**	**+++**	**+++**	**+**	**+++**	**+++**	**++**	−	**+**
**D**	**Serum**	**rAra h 2.0201 (1)**	**+++**	**++**	**+++**	**+++**	**+++**	**+++**	**+++**	**+++**	−	**+**
**E**	**Serum**	**rAra h 6.0101-TR (**1**)**	**++**	**+++**	**+**	**+++**	**+++**	**+++**	**+++**	**+**	−/+	**+**
**F**	**Serum**	**rAra h 1.0101 (5)**	−	−	−	−	−	−	−	−	−	**+**
**J**	**Serum**	**nAra h 1 (5)**	−	−	−	−	−	−	−	−	−	**+**
**H**	**Serum**	**nAra h 3 (5)**	−	−	−	−	−	−	−	−	−	**+**
**I**	**Serum**	**Ara h 2 peptide (0.2)**	−	−	−	−	−	−	−	−	−	**+**
**J**	**Ara h 2-IgE**	**rAra h 1.0101 (1)**	−	−	−	−	−	−	−	−	−	**+**
**J**	**Ara h 2-IgE**	**rAra h 2.0201 (1)**	**+**	**+++**	**+++**	**++**	**++**	−	−	**+++**	−	**+**
**J**	**Ara h 2-IgE**	**rAra h 6.0101-TR (**5**)**	**++**	**++**	**+++**	**+++**	**++**	**+++**	−	**++**	−	**+**

### Ara h 1 IgE is not able to mediate Ara h 1–triggered allergic reaction

**Figure 7.**
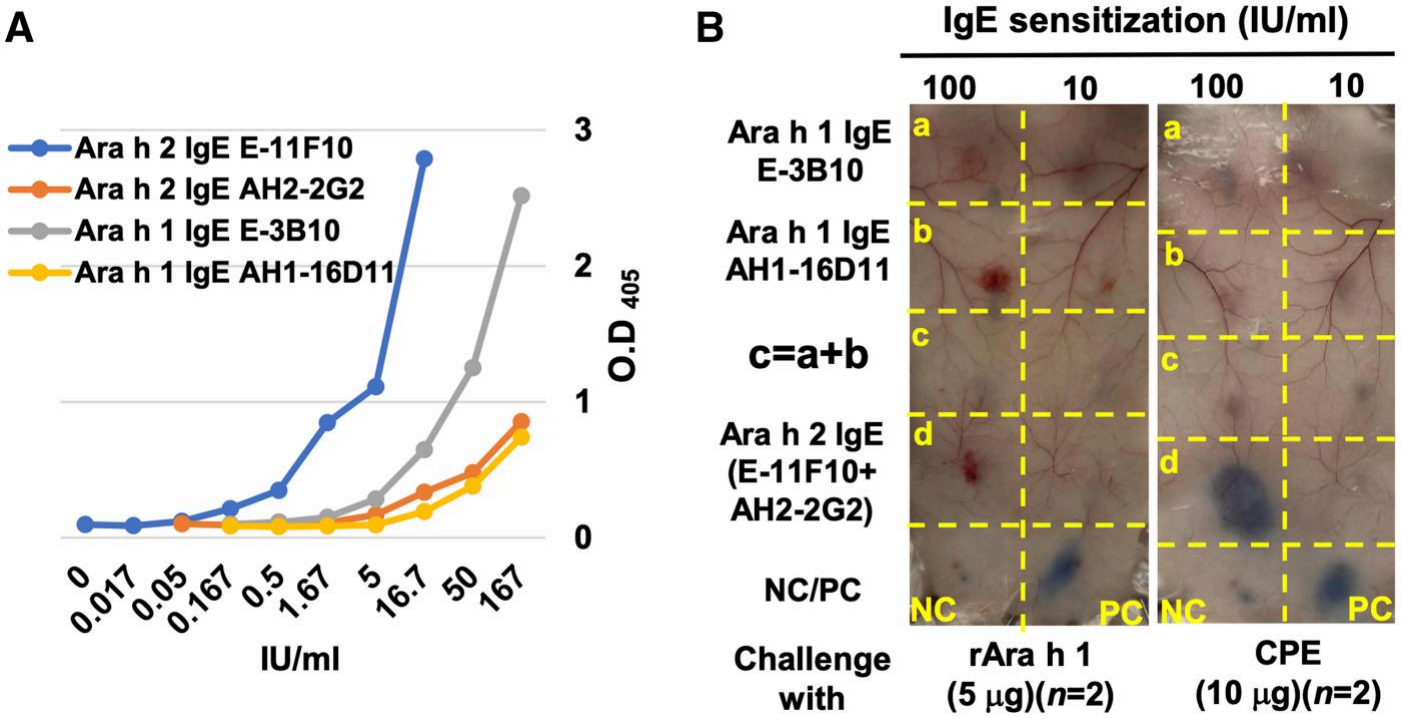
Lack of allergic activity of Ara h 1 IgE in PCA. (A) Binding strengths and kinetics of a pair of Ara h 1 IgE and Ara h 2 IgE to their corresponding peanut allergen components in ELISA. (B) PCA reactions mediated by the sensitized IgE. Mice were locally sensitized with Aar h1 IgE E-3B10 with 100 IU/mL and 10 IU/mL, respectively, at position a, with Ara h 1 IgE AH1-16D11 at position b, with mixture of E-3B-10 plus AH1-16D11 at position c, and mixture of Ara h 2 IgE E-11F10 plus AH2-2G2 at position d for 24 h, followed by challenging the mice with 5 µg rAra h 1 and 10 µg CPE, respectively. NC, negative control; PC, positive control.

Because both nAra h 1 and rAra h 1 were not able to trigger PNA serum–mediated allergic reactions in PCA assay (Fig. 6), we further tested whether Ara h 1 IgE could mediate Ara h 1–triggered allergic reaction in the same PCA model. For this test, 2 mAbs to Ara h 1 were used and combined for the test, as crosslinking of 2 IgE molecules by a single allergen would be needed in order to trigger a typical IgE-mediated allergic reaction. First, the allergen binding ability of the 2 Ara h 1 IgE and 2 Ara h 2 IgE to their corresponding allergens were kinetically determined with ELISA, confirming that they displayed Ara h 1 and Ara h2 binding activity, respectively, although with substantially different binding strengths to their corresponding allergens (Fig. 7A). The 2 Ara h 1 IgE, either individually or combined, at higher (100 IU/mL) or lower (10 IU/mL) concentration, could not mediate PCA reaction either challenged with 5 µg rAra h 1, or 10 µg CPE containing abundant nAra h 1, indicated that 2 different Ara h 1 IgE would not be able to mediate Ara h 1–induced allergic reactions. As a control, a mixture of 2 Ara h 2 IgE could mediate apparent allergic reactions triggered by CPE containing nAra h 2 (Fig. 7B). These results, combined with the data of Fig. 6F and G, further suggested that Ara h 1 did not have allergenicity, and crosslinking of Ara h 1 IgE by Ara h 1 could not mediate detectable allergic reactivity.

## Discussion

IgE cross-reactivity among peanut allergen components has been previously investigated,^9–13^ primarily using ELISA-based approaches. The limitation of these prior studies is that such a potentially consequential phenomenon was not further investigated using different experimental means to achieve additional lines of evidence for confirmation. WB has been an indispensable tool identifying allergen-interactive IgE involving allergic reactions. However, when it comes to analyzing peanut allergen binding IgE from serological samples, high background caused by nonspecific binding often makes interpreting the results a major challenge. Our modified WB protocol using soy proteins as blocking reagent^28^ achieves specific detection of peanut protein component–binding IgE, including clear IgE binding signals to 2 Ara h 2 isoforms from PNA sera. We observed that IgE of PNA bound to a large swath of peanut proteins as smeared signals, ranging from ∼10 kDa to larger than 180 kDa in size. As currently identified 18 peanut proteins capable of binding IgE of PNA were in the MW ranging from 8 kDa (Ara h 12 and Ara h 13)^4^^,^^33^ to 64 kDa (Ara h 1),^34^ the high MW IgE binding species larger than Ara h 1 most likely were the peanut protein complexes formation, such as Ara h 1 trimer formation, Ara h 3 oligomer formation,^4^^,^^34^ or other types of homo- or heteroprotein complex formation, as these high MW IgE binding species completely disappeared under reducing conditions.

The peanut IgE cross-inhibition study using the modified WB approach revealed that rAra h 1, nAra h 3, and/or rAra h 6 could slightly inhibit PNA IgE bound to their corresponding allergens as well as to the broad peanut protein spectrum, whereas rAra h 2 not only substantially inhibited peanut reactive IgE bound to Ara h 2, but also concomitantly blocked IgE binding to almost all fractionated peanut proteins including previously characterized major peanut allergens. These findings indicate that Ara h 2–specific IgE could cross-react with a range of peanut proteins including Ara h 1, Ara h 3, and Ara h 6, revealing that Ara h 2–specific IgE is most likely responsible for IgE cross-reactivity seen with PNA serum. If Ara h 1, Ara h 3, or Ara h 6 were bona fide allergens capable of eliciting rigorous allergen-specific IgE responses as the Ara h 1, Ara h 3, and Ara h 6 IgE levels seen in PNA, one can expect that those Ara h 1–, and/or Ara h 3–specific IgE would not be substantially inhibited by rAra h 2 because they only share a low level of sequence homology. Profound blocking of IgE signals to broad peanut proteins/allergens by rAra h 2.0201 revealed that Ara h 2 IgE likely was the origin of the IgE cross-reactivity among peanut allergens/proteins. Such a notion was further supported by evidence that affinity-purified Ara h 2 IgE, human Ara h 2 IgE mAbs, and mouse anti-Ara h2 mAbs could either bind to the peanut protein compounds other than Ara h 2 itself, or mimic the broad IgE binding patterns of PNA IgE, demonstrating that antibodies against the epitopes presented in Ara h 2 have the propensity to binding to other peanut proteins. Taken together, the available evidence indicated that IgE signals bound to Ara h 1, Ara h 3, Ara h 6, and beyond were most likely derived from Ara h 2–specific IgE through cross-reactivity.

Partial inhibition of IgE bound to Ara h 1, Ara h 3, and Ara h 6 by rAra h 2.0201 in some samples could be attributed to the presence of Ara h 2 IgE with relatively low affinities, as some of the polyclonal Ara h 2 IgE of PNA would not expect to have equal affinities to Ara h 2. Under such situations, the Ara h 2 IgE with lower affinities could not be totally blocked even using high concentration of rAra h 2, as lower-affinity Ara h 2 IgE would be constantly on and off the blocking rAra h 2.0201, leaving them available to bind to the fractionated peanut proteins/allergen on WB, resulting in the observed partial inhibition. Thus, a partial IgE inhibition pattern might be the presence of lower-affinity Ara h 2 IgE, not necessarily indicative of the existence of IgE species specific to the corresponding peanut proteins/allergens.

Analysis of Ara h 1–, Ara h 2–, Ara h 3–, and Ara h 6–specific human IgE derived from naturally occurred PNA revealed that all of them exhibited broad but variable levels of cross-reactivity levels with other peanut allergen components. A noticeable common feature is that all of them could bind to Ara h 2 and Ara h 6, although with different binding strengths (Fig. 4). Their cross-binding ability appeared epitope conformation dependent, as their binding capacity could be eliminated under reducing conditions of allergens from CPE. Analysis of mouse anti-peanut allergen component mAbs also displayed broad cross-reactive patterns among known peanut allergen components (Fig. 5). Taken together, our data indicated that not only Ara h 2, but also other Ara h–specific IgE or mAbs have propensity to cross-react with other components, provided a basis for IgE cross-reactivity seen with PNA.

Allergenicity of Ara h 1 and Ara h 3 were previously tested using cell-based degranulation assay.^8^^,^^35^^,^^36^ The peanut allergic IgE sensitized allergic effector cells were stimulated with purified nAra h 1 or nAra h 3 to demonstrate their ability to trigger degranulation. These experiments generally yielded low levels of activation/degranulation compared with that of Ara h 2.^8^^,^^35^^,^^36^ In some cases, up to several-hundred-fold higher protein concentration than that of Ara h 2 was attempted to examine the allergic reactivity and/or cross-inhibition.^9^^,^^31^^,^^32^^,^^37^ There were caveats using these in vitro functional assays to determine the allergenicity of Ara h 1/Ara h 3, as the purified Ara h 1 and/or Ara h 3 prep might have been contaminated with low levels of Ara h 2/Ara h 6^15^ that were sufficient to trigger degranulation. Such a scenario also could occur in the basophil activation test determining the allergenicity of Ara h 1 and/or Ara h 3. Therefore, the results derived from the cell-based functional assay and/or basophil activation test might not reliably reflect the genuine allergenicity of Ara h 1 and/or Ara h 3 by using purified components derived from natural peanut proteins.

Lack of peanut allergic reactivity triggered by Ara h 1 and Ara h 3 in PCA appeared indicating that these previously characterized major peanut allergens might merely be a group of peanut proteins cross-reacting with Ara h 2 elicited IgE but unable to trigger an allergic reaction via crosslinking and therefore lack true allergenicity. If such a scenario is true, then Ara h 1 and/or Ara h 3 are not qualified as bona fide peanut allergens, as the definition of an allergen should be the protein capable of eliciting a rigorous allergen-specific IgE response, as well as capable of triggering allergic reactivity through crosslinking of the sensitized IgE on allergic effector cells.

Ara h 6 and Ara h 2 are evolutionary related proteins that share ∼55% identity of the overall sequences; 68% identity if the 26 amino acid hypervariable loop sequences of Ara h 2 that was already deleted in Ara h 6 was removed for homologous comparison; and 79% identity in their C-terminal portion sequences.^4^^,^^10–12^ High sequence identity provided the basis for Ara h 6 triggering Ara h 2 IgE-mediated peanut allergic reactions via cross-reactivity. Such a possibility was confirmed as allergic reactions of the affinity-purified Ara h 2 IgE sensitized FcεRIα transgenic mouse could be triggered with rAra h 6-TR challenge. Because Ara h 6 binding IgE was frequently presented in PNA, but monosensitization of Ara h 6 IgE was rarely found, it was likely that in the real PNA settings, Ara h 6 mainly functioned as a potent cross-reactor to trigger Ara h 2 IgE-mediated peanut reactivity but not necessarily triggering Ara h 6–specific IgE-mediated peanut allergic reactions.

Although our challenging results using 25-amino-acid peptide of Ara h 2.0201 containing repeated DPYSPS motifs could not refute the conclusion that the hydroxyproline-containing DPYSP^OH^S motifs are important to Ara h 2’s allergenicity,^30–32^ our results indicate that the repeated linear DPYSPS motifs themselves were not sufficient to trigger peanut allergic reactivity, even though these sequences might involve in IgE binding.^38^ The fact that rAra h 6 was capable of triggering PNA serum and Ara h 2 affinity-purified IgE-mediated PCA reaction also indirectly suggested that the repeated DPYSPS sequence was not important in triggering peanut allergic reaction because these motifs of Ara h 2 were deleted, and therefore did not exist, in Ara h 6. If these repeated motifs were indeed important for the allergenicity of Ara h 2, they might participate in the formation of the structures with other sequences to form conformational epitope(s) to trigger peanut allergic reactions.

As up to ∼15-fold-higher mole level of rAra h 1.0101 (and nAra h 1) than that of rAra h 2.0201 for challenging the PNA allergic IgE sensitized animal could not demonstrate the allergenicity of Ara h 1, and crosslinking of 2 Ara h 1 IgE by both rAra h 1 and nAra h 1 from CPE could not trigger detectable allergic reactions, we concluded that Ara h 1 lacks true peanut allergenicity; therefore, it is unlikely to be a bona fide peanut allergen, but rather is merely a peanut protein capable of cross-reactivity with Ara h 2 IgE. Therefore, it would not likely play a pathological role for clinical PNA. Such a conclusion appears to be reminiscent of the previous finding that peanut allergenicity was not altered in a strain of peanut naturally deficient of Ara h 1.^39^

It remains possible that Ara h 1 (or Ara h 3) could be a peanut allergen with a very weak allergenicity that required even higher concentration in order to trigger peanut allergic reactions in the mouse model, or the current mouse model is not sensitive enough to detect the weak peanut allergenicity. However, such a potential extremely weak allergenicity of Ara h 1/Ara h 3, even if it existed, would unlikely have clinical consequence in real clinical settings of PNA, as mono Ara h1 and/or Ara h 3 IgE sensitization cases were not convincingly confirmed in PNA after decades of research. Under multi-IgE sensitization situations, the potential weak allergic reaction triggered by Ara h1/Ara h3 would be irrelevant as allergic effects derived from Ara h 2 IgE must be dominant and responsible for the overall allergic reactions in PNA. A previous study showed that rAra h 1 displayed much weaker allergenicity compared with its natural counterpart,^40^ but whether the allergenicity of the purified nAra h 1 allergen used in the study was derived from the contamination of Ara h 2/6 in the Ara h 1 preparation was not examined and could not be excluded, based on a recent study.^15^

**Figure 8.**
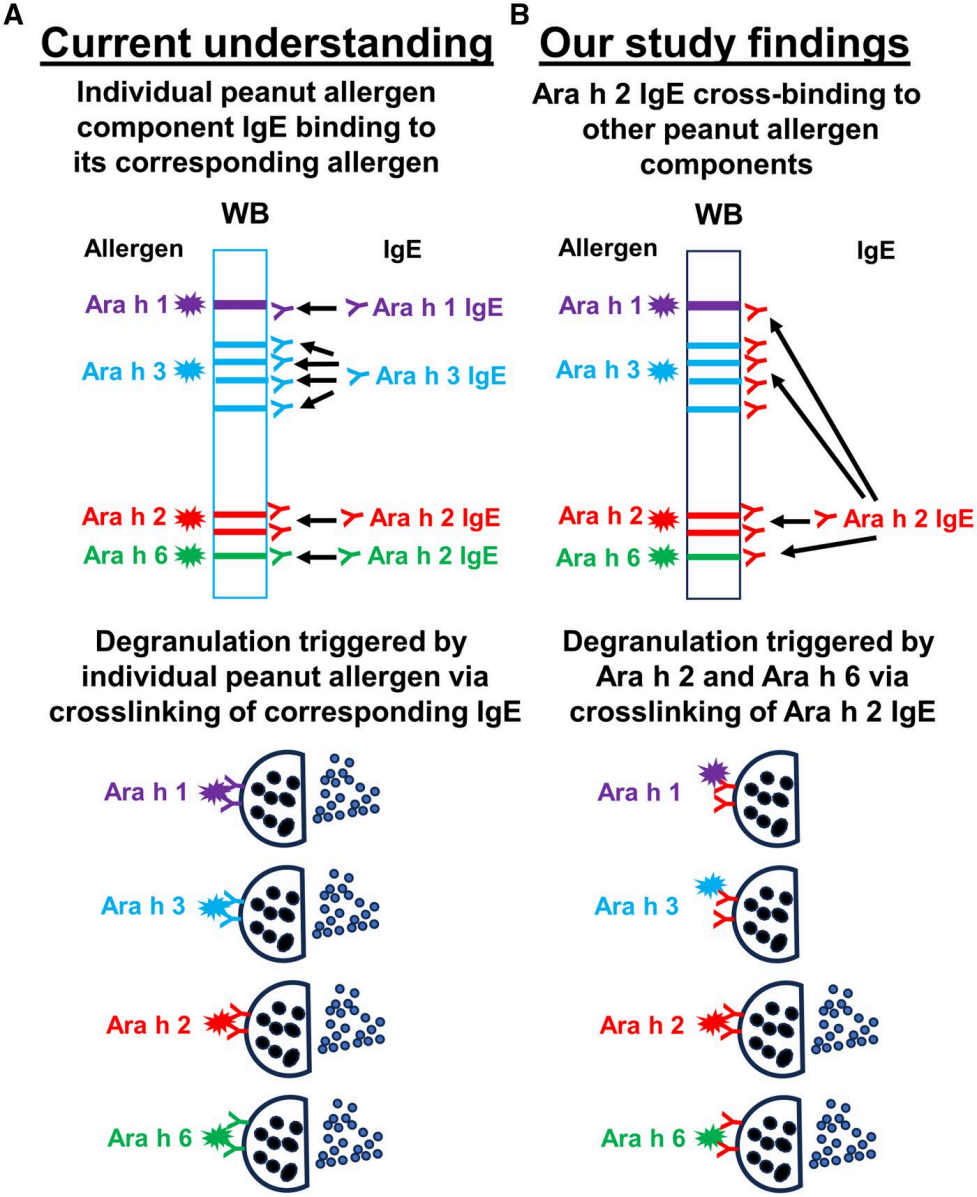
Graphic summary of the findings from the current study. (A) Current understanding of the peanut allergen component IgE and IgE-mediated peanut allergic reactions as the mechanism of PNA. (B) Graphic summary of the findings from the current study. Ara h 2 IgE appears to be the master IgE capable of binding to Ara h 1, Ara h 2, Ara h 3, and Ara h 6; Ara h2 and Ara h 6, but not Ara h 1 and Ara h 3, are able to trigger Ara h 2 IgE-mediated peanut allergic reactions.

Taken together, this study revealed that Ara h 2 IgE is capable of binding multiple peanut proteins including the major peanut allergens Ara h 1, Ara h 3, and Ara h 6, in addition to Ara h 2, indicating that Ara h 2 IgE is responsible for the broad IgE cross-reactivity among peanut allergens/proteins observed in PNA. We further provide evidence that both Ara h 1 and Ara h 3 lacked peanut allergenicity, as they were not able to trigger peanut allergic reactions. Ara h 6 possessed the capacity to function as an allergen to trigger peanut allergic reaction likely by triggering Ara h 2 IgE-mediated peanut allergic reactions through cross-reactivity with Ara h 2 IgE via structural similarities/sequence identities. Figure 8 presents a graphic summary for the current study. This study raises questions contradicting the current understanding of peanut allergens and the mechanisms of PNA, which might impact the future research of PNA allergen immunotherapy and diagnosis.

## Data Availability

All the original data, images, files and raw data will be available upon request.
